# Influence of Immediate Versus Delayed Loading on Peri-Implant Bone Healing: A Comparative FEA Study of Titanium Threaded and Scaffold Dental Implants

**DOI:** 10.3390/ma19081607

**Published:** 2026-04-16

**Authors:** Giuseppe Casalino, Mario Ceddia, Nicola Contuzzi, Luciano Lamberti, Bartolomeo Trentadue

**Affiliations:** Department of Mechanics, Mathematics and Management, Polytechnic University of Bari, 70125 Bari, Italy; giuseppe.casalino@poliba.it (G.C.); nicola.contuzzi@poliba.it (N.C.); luciano.lamberti@poliba.it (L.L.); bartolomeo.trentadue@poliba.it (B.T.)

**Keywords:** dental implant, finite element analysis, porous implant, stress shielding

## Abstract

Background: Immediate loading of dental implants shortens treatment time and improves early function, but it also exposes the healing peri-implant tissue to a critical mechanical environment. This study compared the biomechanical and mechanobiological response of a conventional threaded implant and a porous scaffold-based implant under immediate and delayed loading conditions. Methods: A three-dimensional finite element model of a bone block with a 0.2 mm peri-implant callus was developed in ABAQUS/Standard. Model A was a threaded Ti-6Al-4V implant, while Model B was a porous implant with 64.26% porosity. Bone tissues were modeled as poroelastic materials. Immediate and delayed loading were simulated through frictional and tied bone-implant interfaces, respectively. Mechanobiological predictions were performed using the Prendergast-Huiskes stimulus. Results: Under immediate loading, the porous implant reduced cortical bone stress (32.5 MPa vs. 88 MPa) and markedly increased callus stimulation (20.5–31.6 MPa vs. about 2.5 MPa) compared with the threaded implant. Mechanobiological analysis showed that Model B promoted higher fractions of immature and mature bone and lower fractions of cartilage and fibrous tissue. In all cases, implant stresses remained below the yield strength of the corresponding materials. Conclusions: The porous implant provided a more favorable mechanical environment for early peri-implant healing, particularly under immediate loading, and may be a promising strategy to enhance callus maturation and reduce stress shielding.

## 1. Introduction

The two-stage dental implant placement protocol introduced by Brånemark (1983) requires implants to remain submerged and unloaded for a healing period of approximately three to six months, followed by a second surgical procedure to uncover the implant before prosthetic rehabilitation [1]. In contrast, the immediate loading protocol, in which the restoration is delivered at the time of surgery, significantly reduces both treatment time and the number of surgical interventions [2]. Clinical studies have reported satisfactory survival rates for immediately loaded implants [3,4]. However, the risk of failure remains relatively high when the host site is compromised or when the implant is placed in high-load regions, such as posterior molar sites [5].

Several studies have suggested that peri-implant healing is influenced not only by the timing of loading but also by load magnitude. Esaki et al. [6] demonstrated that a moderate lateral load of 10 N promoted higher bone-to-implant contact and greater bone density than an excessive lateral load of 50 N, which was instead associated with reduced new bone formation. Similarly, Nagasawa et al. [7] showed in a rat model that implant overload may induce degenerative changes in osseointegration and bone loss even in the absence of infection, confirming the potentially detrimental effect of excessive loading during the healing phase. These findings support the view that the mechanical environment established during the early stages after implantation plays a crucial role in determining the biological outcome at the bone–implant interface.

One of the biomechanical mechanisms most frequently associated with implant-related complications is stress shielding, whereby a relatively stiff implant carries an excessive portion of the applied load, thereby reducing the mechanical stimulation experienced by the peri-implant bone [8,9]. This reduction in physiological strain may impair bone maintenance and remodeling, ultimately compromising long-term implant stability [10,11,12]. To mitigate stress shielding, several strategies have been proposed, including the use of implant materials with an elastic modulus closer to that of native bone [13]. A better match between the mechanical properties of the implant and those of the surrounding tissue enables a more physiological load transfer, promotes a more uniform stress distribution, and reduces the likelihood of stress shielding [14].

In recent years, porous metallic implants have attracted considerable attention in implant dentistry because their interconnected pore networks provide a large surface area for bone attachment and facilitate tissue ingrowth [15]. High porosity may promote internal bone formation, thereby improving osseointegration and overall stability [16]. In addition, the effective stiffness of porous structures can be tailored to approach that of human bone by controlling porosity and architectural parameters [17].

Ti-6Al-4V has been extensively used in bone tissue regeneration applications because of its favorable combination of biocompatibility, corrosion resistance, and mechanical strength. Beyond conventional dense implants, porous and additively manufactured Ti-6Al-4V structures have been increasingly investigated as bone-regenerative devices capable of promoting osteoconduction, bone ingrowth, and improved load transfer. Arabnejad et al. [18] reported high bone integration potential in porous titanium structures, while Xiong et al. showed that Ti-6Al-4V scaffolds with a dense core and porous outer layer may combine enhanced osseointegration with adequate fatigue resistance. In vivo evidence has also highlighted the relevance of pore architecture, as Taniguchi et al. [19] demonstrated that pore size significantly affects bone ingrowth into additively manufactured porous titanium implants. More recently, bilayer and spatially distributed porous titanium implant concepts have further supported the interest in Ti-based regenerative implant designs aimed at improving peri-implant healing and long-term stability [20].

Recent advances in metal additive manufacturing (AM), particularly selective laser melting (SLM), have enabled the fabrication of porous implants with highly controlled architectures and reproducible mechanical properties [20,21]. Triply periodic minimal surface (TPMS) lattices, such as gyroid and diamond structures manufactured by laser powder bed fusion (PBF-LB/M), can achieve high porosity and pore sizes comparable to those of trabecular bone [22]. This architectural similarity allows the effective elastic modulus of the implant to be tuned toward values closer to those of human bone, thus improving load sharing and potentially reducing stress shielding.

Despite these advantages, successful dental implant therapy still depends on the maintenance of implant stability within the host bone site, a condition achieved through osseointegration, which includes both healing and remodeling phases [23]. The surgical trauma caused by implant placement initiates a bone healing process characterized by a reparative phase in which mesenchymal stem cells differentiate into various progenitor cell lineages, leading to the formation of fibrous tissue, cartilage, and bone [24]. During peri-implant bone healing, osteoprogenitor cells derived from the bone marrow and endosteal bone surfaces migrate into the healing callus, where they proliferate and differentiate into osteoblasts. These cells begin to deposit new bone onto the existing bone surface and onto the implant surface itself. Woven bone forms rapidly as a temporary scaffold to bridge the gap and provide initial structural support [25,26,27,28].

Careful control of loading conditions throughout this process is essential for successful bone formation. If the mechanical environment is unfavorable, soft tissue may develop instead of mineralized tissue, resulting in poor mechanical stability and possible implant loosening, which is a common sign of failure [29]. Although the biological processes occurring at the bone–implant interface have been extensively investigated experimentally, only a limited number of studies have directly compared the effects of immediate and delayed loading in porous dental implants.

In this context, the present study uses the finite element method (FEM) to evaluate the biomechanical response of two dental implant designs, namely a conventional threaded implant and a porous scaffold-based implant, under two different loading protocols: immediate loading (IL) in a post-extraction condition and delayed loading (DL) after full tissue mineralization. By comparing the mechanical response of these two implant configurations under different healing scenarios, this study aims to provide clinically relevant insights into implant selection and loading strategy, while also contributing to the understanding and mitigation of biomechanical complications such as stress shielding.

## 2. Materials and Methods

### 2.1. Modelling

In recent decades, the finite element method (FEM) has become a reference tool in dentistry for analyzing stress and strain distribution in prosthetic components and peri-implant bone, thus supporting the development and optimization of implant systems [30]. In this study, a static structural finite element analysis was conducted in ABAQUS/Standard 2017 (Simulia, Dassault Systemes, Johnston, RI, USA) to evaluate the effect of the biomechanical response of two dental implant designs (threaded implant and a porous scaffold-based implant).

**Figure 1:**
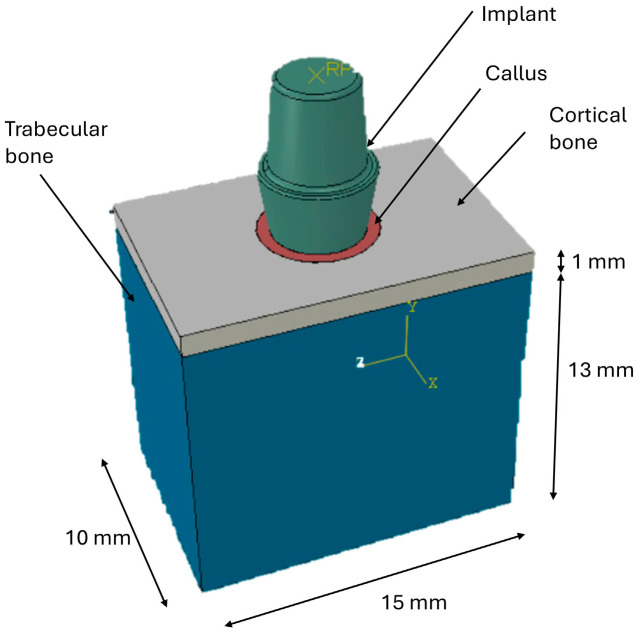
3D model of the bone block and the implant.

A bone block was modeled in Autodesk Inventor 3D CAD (2024), with the dimensions shown in (Figure 1). To reproduce the healing condition, a 0.2 mm-thick peri-implant bone callus was modeled around the implant [31].

Subsequently, two implant configurations were designed using Autodesk Inventor 3D CAD (2024). The threaded implant (Model A) consisted of an endosseous portion 10 mm in length and 4.1 mm in diameter, together with a 7 mm-long coronal portion not in contact with the surrounding bone [32]. The geometry of Model A was selected to represent a conventional threaded dental implant configuration commonly adopted in clinical practice and frequently used in previous numerical studies. Accordingly, this model was used as a reference baseline for comparison with the porous scaffold-based implant.

**Figure 2:**
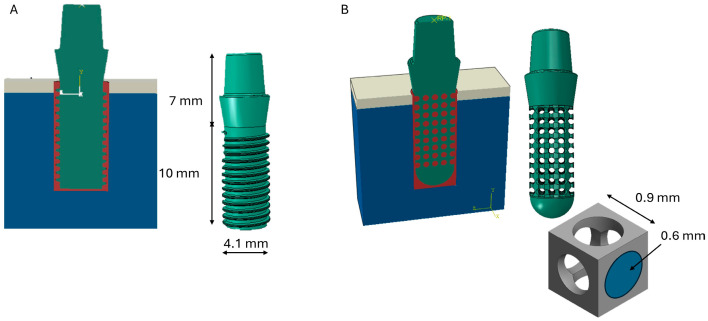
Three-dimensional representation of the implant models analyzed in the present study: (**A**) threaded implant; (**B**) porous implant.

The porous implant (Model B) was characterized by a porous central region, modeled using a scaffold architecture based on a cubic unit cell with a cylindrical pore 0.6 mm in diameter and 0.9 mm in length (Figure 2). The selected pore size and overall porosity were chosen to achieve a balance between mechanical competence and biological performance. Specifically, the cylindrical pore diameter of 0.6 mm (600 μm) falls within the range commonly reported as favorable for bone ingrowth, angiogenesis, and osteogenesis in porous titanium implants. In addition, the adoption of a cylindrical pore geometry was supported by Xu et al. [33], who reported improved compressive and shear stiffness, more uniform stress distribution, greater torsional stiffness, and better hydraulic permeability compared with spherical pores at equivalent porosity. The overall porosity of 64.26% was calculated from the actual porous architecture designed within the implant. At the same time, this level of porosity preserves adequate structural strength for dental implant applications [34].

### 2.2. Mechanical Properties

For smooth and threaded implants, the titanium alloy Ti-6Al-4V was adopted and modeled as a linear elastic, homogeneous, and isotropic material. Under these assumptions, the constitutive behavior was defined by two elastic constants, namely Young’s modulus (E) and Poisson’s ratio (ν).

The porous implant (Model B) was modeled with an explicit porous architecture based on a cubic unit cell. Therefore, the effect of porosity was directly represented in the implant geometry. The solid portion of the scaffold was assigned the mechanical properties of bulk Ti-6Al-4V. The Gibson–Ashby model [35] was used only to estimate the equivalent apparent elastic modulus and yield strength of the overall porous structure for comparative purposes, and not as constitutive properties assigned to the explicitly modeled lattice:

(1) $$E=E_{s}{\left(1-\phi \right)}^{2}$$

(2) $$\sigma =\sigma s{\left(1-\phi \right)}^{3/2}$$

where $E_{s}$ is the elastic modulus of solid titanium (110 GPa), $\sigma_{s}$ is the compressive yield strength of solid titanium (890 MPa), and ϕ is the porosity [36].

For a porosity of 64.26%, the porous titanium structure showed an apparent elastic modulus of 14.05 GPa and a compressive yield strength of 190.16 MPa. Poisson’s ratio of 0.3 was assumed for both implant configurations.

The cortical bone, trabecular bone, and peri-implant callus were modeled as isotropic linear poroelastic materials.

This assumption is consistent with previous mechanoregulation-based numerical studies on peri-implant healing, in which bone and healing tissues were modeled as poroelastic media in order to capture the coupled effects of matrix deformation and interstitial fluid flow on tissue differentiation [37,38,39]. This approach is particularly appropriate when the Prendergast–Huiskes stimulus is adopted, since the evaluation of both octahedral shear strain and interstitial fluid velocity is required within the healing region. Therefore, in the present study, an isotropic linear poroelastic formulation was selected as a computationally efficient and literature-supported approximation of the early biomechanical environment around the implant [25,40].

For the delayed-loading configuration, the 0.2 mm peri-implant region was assigned the mechanical properties of cancellous bone rather than granulation tissue, in order to represent the healed condition after tissue mineralization. This assumption reflects the biological replacement of the initial healing callus by newly formed trabecular bone during osseointegration.

The material definition included the elastic modulus, Poisson’s ratio, permeability, void ratio, and the bulk moduli of the porous skeleton and pore fluid, as summarized in Table 1.

### 2.3. Fea Modeling

**Figure 3:**
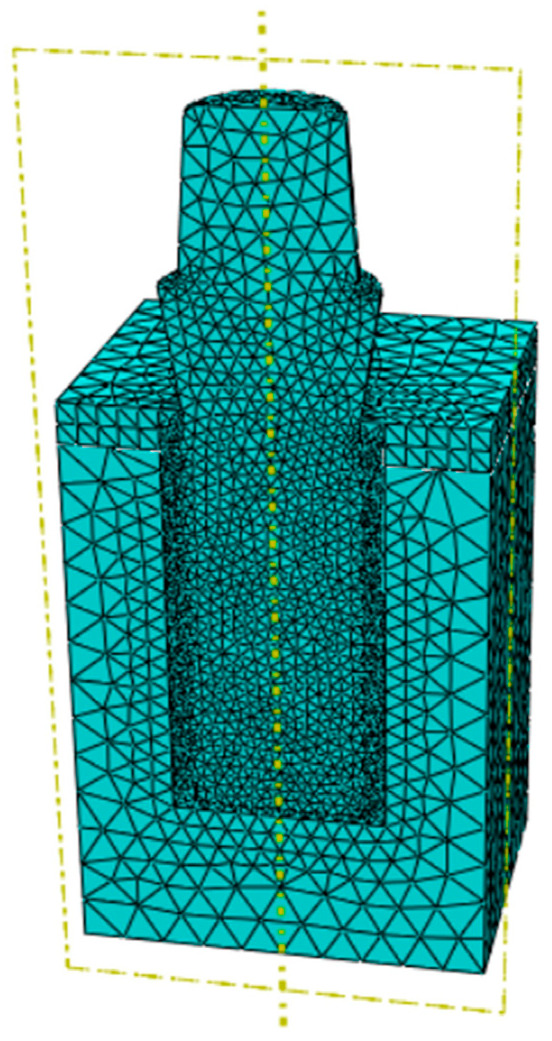
Finite element discretization of the implant and bone block model.

A three-dimensional finite element model was developed in ABAQUS/Standard 2017 (Simulia, Dassault Systèmes). After a mesh convergence analysis, the final mesh configuration included an average element size of 1 mm for cortical and trabecular bone, 0.2 mm for the peri-implant callus, and 0.3 mm for the implant. Cortical bone, trabecular bone, and callus were discretized with quadratic tetrahedral pore-pressure elements (C3D10MP), while the implant was meshed with quadratic tetrahedral elements (C3D10). The final mesh consisted of 20,194 nodes and 79,188 elements for the threaded implant model, and 129,288 nodes and 165,081 elements for the porous implant model (Figure 3).

### 2.4. Boundary Conditions

**Figure 4:**
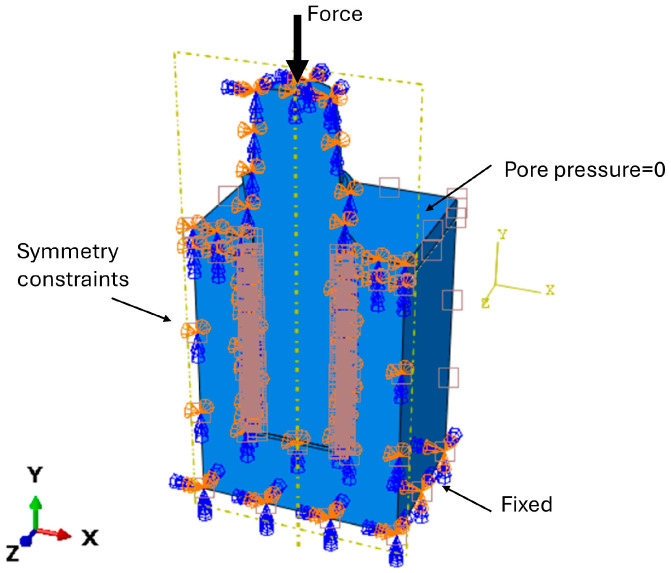
Boundary and loading conditions applied to the analyzed model.

Tie constraints were defined on all contacting surfaces. Due to the geometric symmetry of the model, only a symmetric portion was analyzed, and symmetry boundary conditions (“YASYMM”) were applied on the surfaces lying on the xy plane. The inferior surface of the bone block was fully fixed in all directions, while pore pressure was constrained to zero on the upper surfaces of the cortical bone and peri-implant callus. A vertical load of 100 N was applied at the top of the implant (Figure 4) [25]. To simulate the two healing scenarios, different interface conditions were assigned at the bone–implant interface: a tie constraint for delayed loading (DL), representing complete osseointegration, and a frictional contact with a coefficient of friction of 0.3 for immediate loading (IL), allowing compressive load transfer only [41]. The selected value was based on previous finite element studies in which a friction coefficient of 0.3 was adopted to model the early mechanical interaction at a non-osseointegrated bone–implant interface under immediate loading. Although this parameter may vary according to bone quality, implant surface roughness, and local interfacial conditions, it was considered a reasonable literature-based approximation for the initial healing stage [25,40,41,42,43].

## 3. Results

### 3.1. Stress Distribution in Bone

**Figure 5:**
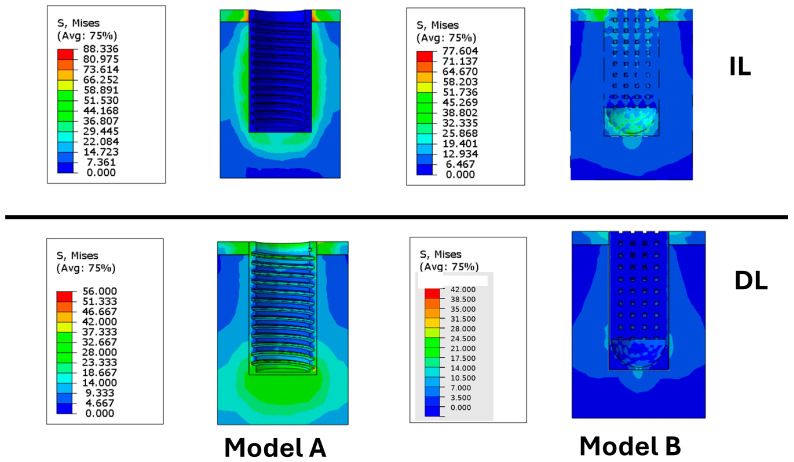
Comparison of equivalent stress distribution in the bone with different implant models under different loading protocols. The (**upper panels**) show the implants subjected to immediate loading (IL), while the (**lower panels**) show those subjected to delayed loading (DL).

The equivalent stress distributions showed clear differences between the delayed loading (DL) and immediate loading (IL) conditions (Figure 5).

Under IL, the highest equivalent stresses were mainly localized in the cortical bone around the implant neck, while lower stress levels were observed in the trabecular bone surrounding the implant threads. In Model A, the maximum equivalent stress in the cortical bone reached approximately 88 MPa, while in Model B it was markedly lower, at about 32.5 MPa.

A substantial difference between the two configurations was observed in the mechanical stimulation of the bone callus. In Model A, corresponding to the threaded implant, the callus was only minimally stimulated under IL, with nearly constant stress values of about 2.5 MPa. By contrast, Model B, representing the porous implant, induced markedly higher stress levels within the callus, with values of approximately 20.5 MPa in the upper region and up to 31.6 MPa in the lower region. These results indicate that the porous implant promoted substantially greater mechanical stimulation of the healing tissue than the threaded design.

Under DL condition, both models generally exhibited lower stress levels than those observed under IL. In Model A, relatively high stresses were still found in the cortical region and at the implant apex, reaching approximately 34.7 MPa and 37.6 MPa, respectively. In this case, the callus stress ranged from 9 to 26.5 MPa, indicating a more effective load transfer to the healing tissue after tissue maturation. In Model B, lower stress values were observed overall, with approximately 16 MPa in the cortical bone and callus stresses ranging from 2.26 to 7.56 MPa.

**Figure 6:**
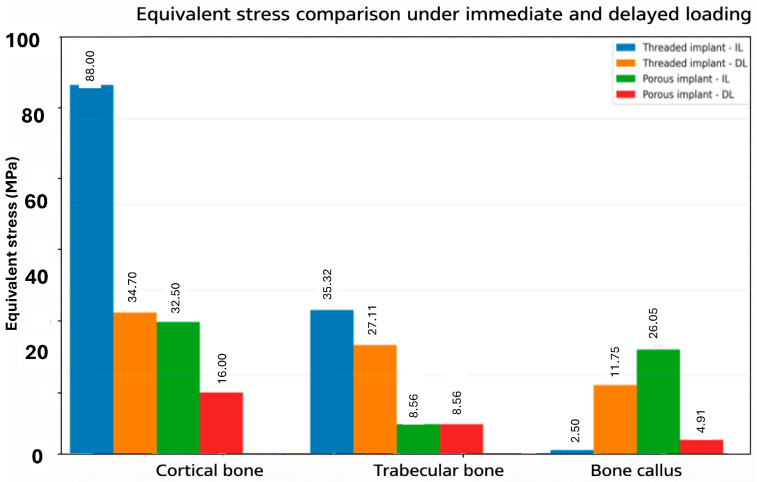
Distribution of equivalent stress in the peri-implant bone tissues for the two implant models under immediate and delayed loading conditions.

To further clarify the differences in equivalent stress distribution among the investigated models, the corresponding values for cortical bone, trabecular bone, and bone callus were summarized in the bar chart shown in (Figure 6).

Overall, the results showed that both the loading protocol and implant design affected the stress distribution within the peri-implant tissues. Under immediate loading, Model B reduced cortical bone stress by approximately 63% compared with Model A (32.50 MPa vs. 88 MPa), while the stress within the peri-implant callus increased from 2.50 MPa to 26.05 MPa.

Although the porous implant exhibited lower cortical stress peaks and a different load-transfer pattern, these findings suggest a more favorable redistribution of load within the peri-implant tissues, which may be associated with a reduced tendency toward stress shielding.

### 3.2. Stress Distribution on the Implants

**Figure 7:**
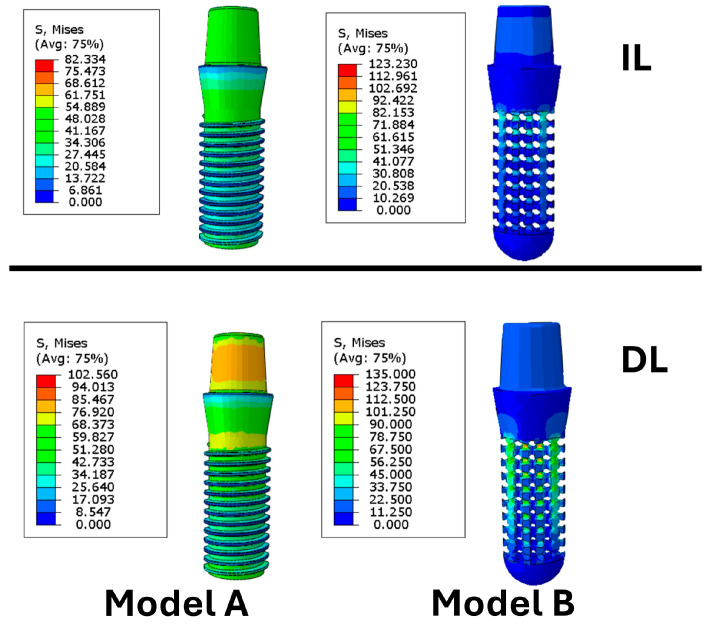
Equivalent stress distribution in implant models under different loading protocols. The upper panels show implants subjected to immediate loading (IL), and the lower panels show those subjected to delayed loading (DL).

Regarding the equivalent stress developed within the implants, the highest values were observed under delayed loading conditions. As shown in (Figure 7), maximum equivalent stresses of 75 MPa and 85 MPa were recorded for Model A and Model B, respectively. Under immediate loading, lower stress values were observed, with maxima of 38 MPa for Model A and 48.56 MPa for Model B.

Considering the yield strength of the implant materials, equal to 890 MPa for the threaded implant (Model A) and 190.16 MPa for the porous implant (Model B), the recorded stress values remained well below the respective critical thresholds. Therefore, under all the investigated loading conditions, neither implant exhibited stress levels indicative of structural yielding.

### 3.3. Mechanobiological Response of the Bone Callus Under Immediate Loading Condition

The mechanobiological tissue-differentiation analysis was performed only for the immediate loading condition, since tissue differentiation mainly takes place during the early healing phase, when the peri-implant region is still occupied by immature healing tissue. In contrast, the delayed loading condition was intended to represent a later stage of healing, in which the peri-implant region had already undergone mineralization and had been replaced by newly formed bone. Therefore, in this case, the analysis focused on the biomechanical response rather than on tissue differentiation.

The Prendergast–Huiskes model was selected because it is one of the most widely used mechanobiological frameworks for predicting early skeletal tissue differentiation and has been extensively applied in studies of peri-implant healing [44,45]. Unlike strain energy density-based remodeling models, which are mainly used to describe bone density adaptation during the remodeling phase, the Prendergast–Huiskes approach is specifically intended for the analysis of early tissue differentiation. Moreover, compared with mechanobiological models based primarily on solid-phase mechanical variables, such as the Claes–Heigele model [46], it incorporates both octahedral shear strain and interstitial fluid velocity. This enables the combined effects of matrix deformation and fluid flow on cell differentiation to be taken into account. For this reason, the model was considered particularly suitable for the present study, in which the peri-implant tissues were modeled as poroelastic materials, and the aim was to predict the early healing response of the bone callus under immediate loading conditions. The mechanobiological stimulus, S, was defined as

(3) S=γ/a+v/b

where γ is the octahedral shear strain, v is the interstitial fluid velocity, and a=0.0375 and b=3 are empirical constants. Tissue phenotype was then assigned according to the following criteria: fibrous tissue for S>3, cartilage for 1<S≤3, immature bone for 0.266<S≤1, mature bone for 0.010<S≤0.266, and initial resorption for S≤0.010 [42].

Based on the finite element evaluation of γ and v for each element within the bone callus, the corresponding tissue phenotype distribution was determined according to the above criteria. The resulting values are reported in Table 2.

The mechanobiological analysis highlighted a clear difference between the two implant designs. Compared with the threaded implant (Model A), the porous implant (Model B) promoted a substantially higher proportion of immature and mature bone, together with lower amounts of cartilage and fibrous tissue. This indicates that Model B provided a more favorable stimulus for osteogenic differentiation and callus maturation, while Model A was associated with a healing response still largely dominated by cartilage formation.

## 4. Discussion

Primary stability and osseointegration are essential prerequisites for the clinical success of dental implants [47]. Osseointegration implies direct contact between the implant and the surrounding bone, ensuring stable anchorage without relative motion. Consistent with this concept, several studies have identified the crestal region as the most critical site for early peri-implant bone loss [48,49,50,51,52].

Clinically, implants may be placed according to two main loading protocols: delayed loading and immediate loading [53]. In the delayed-loading protocol, the implant is left unloaded during the healing period to allow osseointegration before prosthetic loading, which is traditionally performed after approximately 3–6 months [54]. By contrast, immediate loading refers to functional loading within 48–72 h after implant placement, or within the first week depending on the adopted classification [55]. Immediate loading has attracted increasing interest because it shortens treatment time and allows earlier restoration of function and esthetics. Susarla et al. [56] highlighted that its main advantages include reduced overall treatment time and immediate recovery of function and appearance. Similarly, Sánchez-Torres et al. [57] reported beneficial effects on patient satisfaction and on the psychological burden associated with edentulism.

Despite these advantages, the mechanical environment generated during immediate loading remains critical, since the tissues surrounding the implant in the early healing phase exhibit much lower mechanical properties than mature bone. Under these conditions, excessive stress and strain transfer may impair bone formation. According to the Prendergast–Huiskes tissue differentiation theory [58], an unfavorable mechanical stimulus may promote fibrous tissue formation rather than mineralized tissue, thereby compromising osseointegration.

Stress transfer is governed by several factors, among which implant material and structural design are particularly important [59,60,61]. Conventional titanium implants, owing to their high stiffness, may concentrate stresses in the crestal region while reducing the mechanical stimulus transmitted to the surrounding tissue. This condition may increase the risk of local microdamage, bone resorption, and stress shielding. In contrast, structures with stiffness values closer to those of bone may promote a more physiological stress distribution within the peri-implant tissues.

In this context, porous implants represent a promising strategy for improving load transfer during healing [62,63,64]. By reducing the apparent elastic modulus of the implant, porous architectures may favor a more physiological redistribution of stress and enhance the mechanical stimulus delivered to the peri-implant tissues. In addition, their increased surface area and pore interconnectivity may promote cell adhesion, bone ingrowth, osteoconduction, and osseointegration [65,66,67,68,69,70,71]. Among the geometric parameters, pore size appears particularly relevant. Taniguchi et al. [19] demonstrated its influence on bone formation and ingrowth, while the literature generally identifies an optimal range of approximately 400–600 μm for angiogenesis and osteogenesis [72]. Mehboob et al. [73] also reported that increasing implant porosity affects micromotion and stress distribution in cancellous bone, depending on the degree of osseointegration. Likewise, Liu et al. [74] showed that a gradient porous structure with a porosity of 59.86% provided favorable mechanical properties, including maximum equivalent stresses below the compressive yield strength of titanium and an elastic modulus closer to that of bone.

The present findings are consistent with this evidence. Under immediate loading, the porous implant produced lower stress peaks in the cortical bone and higher mechanical stimulation within the healing callus than the threaded implant. This trend was further supported by the Prendergast–Huiskes stimulus analysis, which showed that Model B promoted higher proportions of immature and mature bone, together with lower fractions of cartilage and fibrous tissue. These findings suggest a more favorable environment for osteogenic differentiation and callus maturation in the porous implant, whereas the threaded configuration remained associated with a less advanced healing response, still largely dominated by cartilage formation.

Nevertheless, greater mechanical stimulation of the healing callus should not be interpreted as universally beneficial. Bone formation is expected only when the local biophysical stimulus falls within a favorable osteogenic window. Experimental evidence indicates that controlled interfacial micromotion may stimulate bone formation, whereas excessive micromotion or loading may impair osseointegration and promote fibrous tissue formation. Therefore, the effect of mechanical stimulation on peri-implant healing should be regarded as biphasic rather than monotonic. Within the Prendergast–Huiskes framework adopted in the present study, the most favorable stimulus ranges for bone formation correspond to those associated with immature and mature bone formation (0.266 < *S* ≤ 1 and 0.010 < *S* ≤ 0.266, respectively), whereas higher values are associated with cartilage and fibrous tissue. Accordingly, the improved healing response predicted for the porous implant under immediate loading should be interpreted not simply as the result of greater callus stimulation, but rather as the consequence of a more favorable redistribution of the local mechanobiological stimulus toward the osteogenic range.

By contrast, the delayed-loading condition was intended to represent a mineralized and healed peri-implant state. For this reason, in this case the stress distribution was interpreted only from a biomechanical perspective and not in terms of mechanobiological tissue differentiation. Within this later healing scenario, the stress values predicted in the cortical and trabecular bone for both Model A and Model B fell within ranges considered compatible with bone remodeling (20–60 MPa for cortical bone and 6–18 MPa for trabecular bone) [75]. This suggests that, once healing and mineralization have occurred, both implant configurations may provide a mechanical environment compatible with physiological bone adaptation, although with different patterns of load transfer.

### Limitations

This study has several limitations that should be acknowledged. First, cortical bone, trabecular bone, and the peri-implant callus were modeled as isotropic linear poroelastic materials, whereas bone is inherently orthotropic and exhibits direction-dependent mechanical behavior. Therefore, the adopted material model represents a simplification of the actual biological structure. Second, the delayed-loading condition was simulated by assuming perfect osseointegration at the bone–implant interface through a tie constraint, although in vivo osseointegration is a progressive process characterized by different degrees of interfacial bonding rather than complete mechanical continuity. Third, the applied load was simplified as a static axial force, whereas physiological masticatory loads are dynamic, multidirectional, and variable over time. In addition, the present investigation was limited to static analysis and did not include cyclic loading or fatigue assessment. This aspect is particularly relevant for porous Ti-6Al-4V structures, for which fatigue behavior represents a critical factor in structural safety.

Furthermore, as with other phenomenological mechanobiological models, the Prendergast–Huiskes approach relies on literature-based threshold values that may vary depending on the anatomical site, loading mode, and biological conditions. Therefore, the present mechanobiological predictions should be interpreted primarily in comparative terms rather than as absolute patient-specific thresholds.

Finally, although the present numerical findings provide useful biomechanical and mechanobiological insights, they should be further validated through comparison with experimental and clinical data.

## 5. Conclusions

Within the limitations of this finite element study, both the loading protocol and implant design affected stress transfer within the peri-implant tissues. In particular, under immediate loading, the porous implant reduced cortical bone stress and increased mechanical stimulation of the healing region compared with the threaded implant. Model B showed clear biomechanical advantages under immediate loading, with lower cortical stress peaks and greater mechanical stimulation of the healing callus than Model A. This trend was further supported by the mechanobiological analysis, which predicted higher proportions of immature and mature bone and lower fractions of cartilage and fibrous tissue for the porous implant, indicating a more favorable environment for osteogenic differentiation and callus maturation. In all simulated conditions, the stresses developed within both implants remained below the corresponding yield strengths, suggesting adequate static structural safety.

The present findings should be interpreted with reference to the specific loading and interface conditions considered in this study. Future investigations should include oblique and cyclic loading conditions, parafunctional scenarios, and intermediate bone–implant interface conditions representative of partial osseointegration.

## Figures and Tables

**Table 1:** Material properties of bone used in the study.

Tissue	*E* (MPa)	*ν*	Permeability	Void Ratio	Grain/Fluid Bulk Modulus
Cortical bone	20,000	0.30	9.81 × 10^−11^	0.04	13,920/2300
Trabecular bone	6000	0.30	3.629 × 10^−6^	2.33	13,920/2300
Granulation tissue	0.2	0.17	9.81 × 10^−8^	4.00	2300/2300

**Table 2:** Distribution of tissue phenotypes within the bone callus predicted according to the Prendergast–Huiskes mechanobiological stimulus for the two implant configurations under immediate loading conditions. Values are reported as the percentage of callus elements falling within each stimulus range.

Tissue Phenotype	Stimulus Range (S)	Model A—Threaded Implant (%)	Model B—Porous Implant (%)
Fibrous tissue	*S* > 3	2.85	0.54
Cartilage	3 ≥ *S* > 1	50.42	14.46
Immature bone	1 ≥ *S* > 0.266	19.18	35.65
Mature bone	0.266 ≥ *S* > 0.010	27.55	49.16
Resorption	0.010 ≥ *S*	0.00	0.19

## Data Availability

The original contributions presented in this study are included in the article. Further inquiries can be directed to the corresponding author.
